# PfMDR1: Mechanisms of Transport Modulation by Functional Polymorphisms

**DOI:** 10.1371/journal.pone.0023875

**Published:** 2011-09-01

**Authors:** Pedro Eduardo Ferreira, Gabrielle Holmgren, Maria Isabel Veiga, Per Uhlén, Akira Kaneko, José Pedro Gil

**Affiliations:** 1 Malaria Research Lab, Department of Medicine, Karolinska Institutet, Stockholm, Sweden; 2 Institute for Microbiology, Tumour and Cell Biology, Karolinska Institutet, Stockholm, Sweden; 3 Department of Medical Biochemistry and Biophysics, Karolinska Institutet, Stockholm, Sweden; 4 Drug Resistance and Pharmacogenetics Group, Institute of Biotechnology and Bioengineering, Centre of Molecular and Structural Biomedicine, University of Algarve, Faro, Portugal; 5 Unit of Pharmacogenetics, Department of Physiology and Pharmacology, Karolinska Institutet, Stockholm, Sweden; 6 Department of Biological Sciences, The Harpur College of Arts and Sciences, Binghamton University, Binghamton, New York, United States of America; State University of Campinas, Brazil

## Abstract

ATP-Binding Cassette (ABC) transporters are efflux pumps frequently associated with multidrug resistance in many biological systems, including malaria. Antimalarial drug-resistance involves an ABC transporter, PfMDR1, a homologue of P-glycoprotein in humans. Twenty years of research have shown that several single nucleotide polymorphisms in *pfmdr1* modulate *in vivo* and/or *in vitro* drug susceptibility. The underlying physiological mechanism of the effect of these mutations remains unclear. Here we develop structural models for PfMDR1 in different predicted conformations, enabling the study of transporter motion. Such analysis of functional polymorphisms allows determination of their potential role in transport and resistance. The bacterial MsbA ABC pump is a PfMDR1 homologue. MsbA crystals in different conformations were used to create PfMDR1 models with Modeller software. Sequences were aligned with ClustalW and analysed by Ali2D revealing a high level of secondary structure conservation. To validate a potential drug binding pocket we performed antimalarial docking simulations. Using aminoquinoline as probe drugs in PfMDR1 mutated parasites we evaluated the physiology underlying the mechanisms of resistance mediated by PfMDR1 polymorphisms. We focused on the analysis of well known functional polymorphisms in PfMDR1 amino acid residues 86, 184, 1034, 1042 and 1246. Our structural analysis suggested the existence of two different biophysical mechanisms of PfMDR1 drug resistance modulation. Polymorphisms in residues 86/184/1246 act by internal allosteric modulation and residues 1034 and 1042 interact directly in a drug pocket. Parasites containing mutated PfMDR1 variants had a significant altered aminoquinoline susceptibility that appears to be dependent on the aminoquinoline lipophobicity characteristics as well as vacuolar efflux by PfCRT. We previously described the *in vivo* selection of PfMDR1 polymorphisms under antimalarial drug pressure. Now, together with recent PfMDR1 functional reports, we contribute to the understanding of the specific structural role of these polymorphisms in parasite antimalarial drug response.

## Introduction

Efforts to control *Plasmodium falciparum* malaria are currently reliant on vector control and chemotherapy. Unfortunately, the parasite has demonstrated a persistent ability to circumvent antimalarial drug efficacy through resistance-conferring mutations, as most dramatically illustrated by the collapse of chloroquine as worldwide mainstay chemotherapy [1]. Lack of an effective alternative chemotherapy led to a documented rise in the public health impact of malaria and a significant increase in the disease's related mortality [2], [3].

A cornerstone event in malaria chemotherapy occurred in Thailand, during the 1990s: the recovery of the efficacy of mefloquine (MQ) through its combination with artesunate [4], [5]. Following this successful implementation, conceptually similar artemisinin derivative combination therapies (ACT) were progressively adopted worldwide. Consequently, ACT is presently recognised as an absolute central factor in *P. falciparum* malaria control [6]. It has been proposed that *P. falciparum* resistance to ACT could evolve [7], [8]. Indeed, recent reports have provided the first indications that resistance to ACTs may be emerging in natural parasite populations [9], [10]. If such resistance spreads widely, our drug-based efforts to control malaria will be severely held back.

Drug treatments and policies, purposely engineered to avoid the development of Multi-Drug Resistance (MDR) mechanisms are urgently required. This challenge demands a fundamental understanding of the details of the resistance mechanisms utilised by *P. falciparum*.

In higher mammals, the ATP-binding cassette (ABC) superfamily subclass B1, typified by the mammalian P-glycoprotein (Pgp) [11], have been consistently linked to drug-resistance phenotypes in a large range of organisms [12]. *P. falciparum* contains in its proteome a Pgp-homologue (PfMDR1) [13], [14]. Single nucleotide polymorphisms (SNPs) in its coding gene (*pfmdr1*) have been shown to be associated with differential *in vivo* and *in vitro* parasite responses to a significant range of central ACT antimalarial partner drugs, including amodiaquine [15], [16], mefloquine [7], [17]–[19], lumefantrine [8], [20] and, importantly, artemisinin [18], [19]. PfMDR1 is hence considered an important potential candidate for mediating ACT resistance [21], [22]. However the biophysics and mechanistic role of these polymorphisms remains poorly understood.

In the present study we demonstrate 3D structural models for PfMDR1 and merge available, but never fully integrated, molecular/functional data on this transporter and the functional effects of its main polymorphisms, including *in vitro* antimalarial susceptibility.

Recent observations of the effects of the clinical use of different antimalarial upon allele selection are discussed in terms of their contribution to drug resistance and mechanism of action contributing to evidence based view of antimalarial implementation.

## Results

### PfMDR1 structure

PfMDR1 is a member of the ABC protein superfamily. It is a homologue of Human Pgp-1 composed of two symmetric parts (defined as domain 1 and 2, from N-terminal to C-terminal). Each domain has a transmembrane domain (TMD), composed of three external loops (EL) and two internal helixes (IH) that link six transmembrane regions (TM) followed by a nucleotide binding domain (NBD) (Figure S1).

As a reference for our model, we considered the bacterial ABC lipid flippase (MsbA). This transporter is structurally and functionally related to eukaryotic MDR-type proteins [23]. The documented determination of MsbA structures captured in different conformations allows the basis for structural and motion extrapolations for other ABC full transporters [24].

Comparison of the PfMDR1 primary and secondary structure with those of MsbA, revealed the existence of structure/function conservation between the two proteins, reflected by a ∼22% identity in total homology of PfMDR1 aminoacid residue sequence. In addition, the alignment showed a righteous match in the aminoacid type homology and consequently in secondary structure, pointing towards a structural and functional conservation (Figure S1).

The main sequence divergence in homology occurs in a part of the PfMDR1 NBD's. In NBD1 there is an aminoacid frame insertion after the Q-loop and in NBD2 the insertion occurs after the P-loop. Another observed insertion is located in the TM5 and in its equivalent region of the second domain, designated TM10. TM5 and TM10 in PfMDR1 are expected to be longer than those observed to the MsbA structures. The consistence of this divergence in the homologous halves supports the existence of symmetry between the two domains of ABC transporters (Figure S1).

### Structural changes in PfMDR1

**Figure 1:**
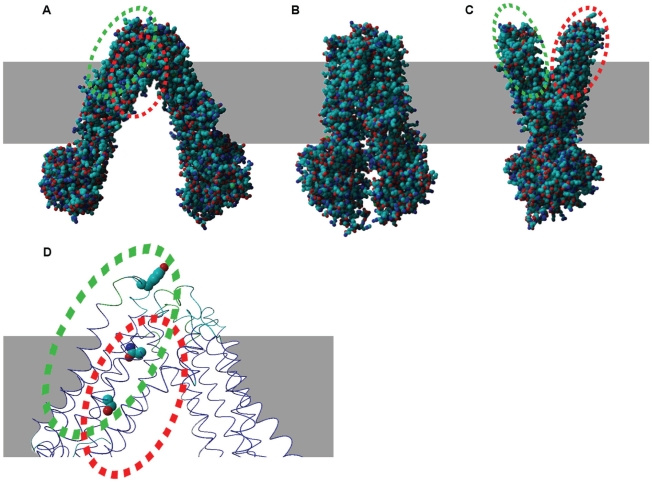
PfMDR1 structure models. PfMDR1 structures are shown in different conformations: inverted V shape (open-apo) based in bacterial MsbA 3B5w structure (A), close-apo based in 3B5x (B) and V shape based in 3B60 structure (C). Panel (D) shows localization of residues 86 (EL1), 1034 and 1042 (TM11) in the open state. Dashed green circle represents residue 86 location and red dashed circle 1034 and 1042.

It has been previously demonstrated that a large range of motion is required for the MsbA transporter to function [24]. For PfMDR1, two hinges whose movement allow for different conformations correspond to EL2 (residue 189–194) and EL3 (residue 311–315) and the equivalent hinge EL5 (residue 929–933) and EL6 (residue 1055–1059) (Figure 1 A–C).

The TMDs communicate with NBDs through IH contact. A particular characteristic is the intertwined interface between the two halves of the transporter, interlocking TM4/TM5/IH2 with NBD2 and TM10/TM11/IH4 with NBD1. Recently, the importance of this structural feature for ABC transporter substrate binding, signalling, and transport was shown. The bonding of the nucleotide transmits a structural change to the TMs via IH's, resulting in an outward-facing conformation activating transport [25], [26].

### *Pfmdr1* single nucleotide polymorphisms: implications for PfMDR1 substrate transport capacity

In Africa, two major PfMDR1 variants are found that differ at 3 aminoacid residues: N86Y, F184Y and D1246Y. The haplotype NFD is associated with decreased parasite sensitivity to arylaminoalcool quinoline drugs (e.g. mefloquine, lumefantrine). Accordingly, selection of this haplotype has been consistently observed during artemether-lumefantrine, (Coartem®, Novatis AG, Basel) treatment [8], [20], [27], whereas the alternative haplotype YYY, is linked to decreased sensitivity to 4-aminoquinoline drugs such as chloroquine and amodiaquine [15], [28]–[31].

EL1 is known to be N-glycosylated in the human multidrug transporter Pgp-1 [32]. Asparagines at position 84 and 86 in the EL1 of PfMDR1 were predicted to be glycosylated with a potential of 0.56 and 0.73 respectively (Figure S2). When we replaced in the sequence 86N residue for the tyrosine variant (86Y), the potential glycosylation site was abolished. However, because of the low *N*-glycosylation capability in *P. falciparum*, its physiological impact remains to be investigated. [33].

For the MsbA transporter, the EL1, which connects TM1 and TM2, is also proposed to be important in open-apo conformation stability through contact with EL6 which links TM11 and TM12. In order to establish a protein nucleotide-bound conformation, the PfMDR1 EL1 and EL6 split and lose interaction transforming the transporter in a V-shape (Figure 1A–C). Indeed, in MsbA, the a.a. residue 56 localized in the EL1 region was shown to interact covalently through spontaneous disulphide bounding with the opposing monomer by cysteine cross-linking experiments in the ATP unbound form [34] demonstrating the proximity between the EL1 and EL6 loops. A similar structural arrangement has been reported to occur in human Pgp, highlighting the importance of TM1 and TM11 terminals at EL's closeness, in ABC transporters function [35].

**Figure 2:**
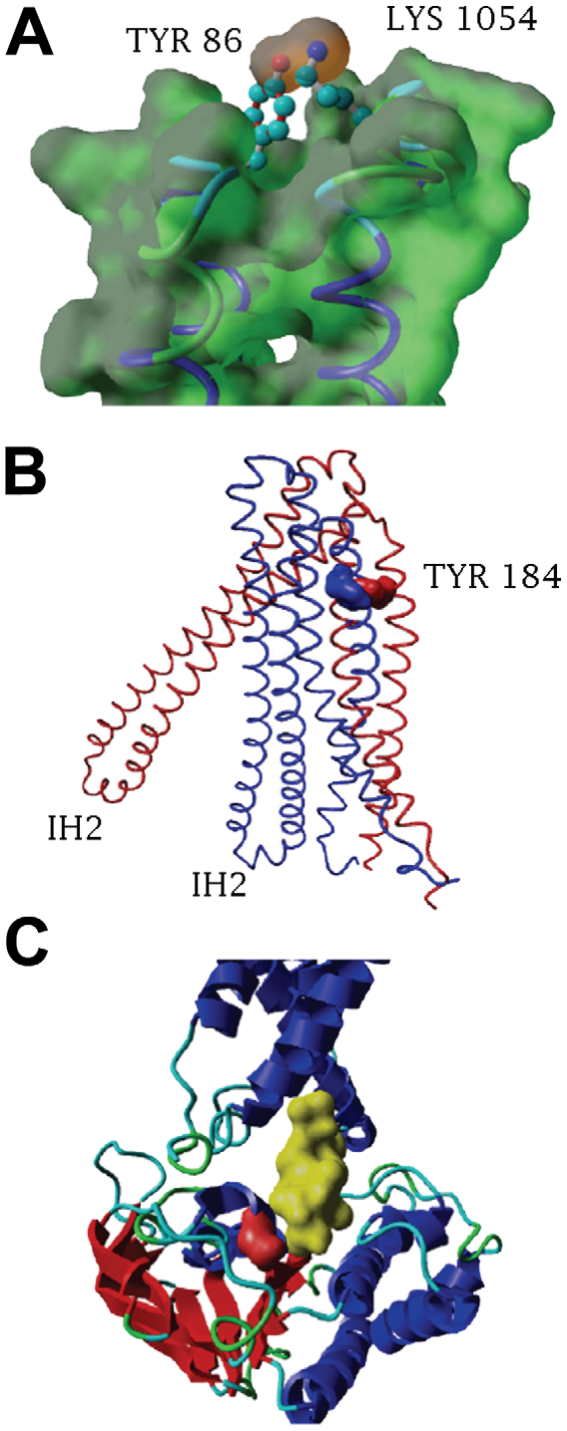
Structural localization of PfMDR1 polymorphisms. **A**) Interaction of EL1-EL6 aromatic side-chain of residue 86Y approximation to the cationic side-chain 1054K in the resting state. Interaction is shown in the Van der Waals surface (dark orange) between the O (red atom) of the 86Y side-chain with N (blue atom) in 1054K side-chain. **B**) Location of residue 184 in TM3 shown in two different conformation structures - open (red) and close (blue). **C**) Location of residue 1246 in NBD2 - Residue 1246 surface is shown in red localized in the cleft interacting with IH2 (yellow aminoacids surface) in the opposed domain.

Analyzing the contact residue in the PfMDR1 EL6 we found that the side-chain of 86Y in EL1 localizes in parallel with residue 1054K (Figure 2A). TM1 and TM11 interactions have been shown in human Pgp to be fundamental for the correct positioning of TMs, while modulating the transporter affinity in a drug specific manner [25], [36]. Accordingly, a significant change in drug affinity caused by the TM1 located N86Y mutation was observed [37] suggesting an analogous biophysical role when residue 86 is mutated to a tyrosine.

PfMDR1 a.a. residue 184 is embedded in TM3, facing the transporter out surface. Analogous to human P-glycoprotein, a mutation in TM3 was shown to alter transport kinetics, independent from drug binding capacity [38]. TM3 is located in the middle of TMD1 surrounded by TM1–TM2 on one side and TM4–TM6 in the other. TM4 and TM5 are closely associated with TM3 in the nucleotide bound conformation (Figure 2B). These major changes, between close and open conformation, are due to the flexibility of EL3 and EL4, which constitute an important hinge in the transporter structure from an “inverted V” shape to a “V” shape and enabling membrane transport interference [24]. In such mobile structures small changes, such as tyrosine to phenylalanine (being both aromatic aminoacids), may cause disturbance in protein dynamics.

The Y184F SNP appears to alter PfMDR1 kinetics but not drug specificity [37], [39] as showed in the *pfmdr1* allelic exchange experiments investigating antimalarial resistance [18]. When compared with the other SNPs in PfMDR1, polymorphism at position 184 exhibit a weaker antimalarial resistance association *in vivo* [7], [20], [40]–[45].

Polymorphisms located in the NBD of ABC transporters were reported to alter protein transport kinetics through ATP hydrolysis capacity alteration [46] or by altering the communication between the NBD and the transmembrane domains [47]. Residue 1246 is located in the NBD2 near the Q-loop and is part of the cleft in NBD2 that interacts with IH2 at the N-terminal (Figure 2C), and is essential for ABC transporter function [48]. PfMDR1 ATPase basal activity was shown not to be blocked by the D1246Y mutation alone. Reduction of PfMDR1 ATPase activity was also reported to occur only when 1246Y is associated with 1034S or 1042D, but not alone [39].

Our model suggests that the functional impact of the D1246Y mutation occurs through interference with the NBD/TM communication, which is required for ABC transporter function. Furthermore, this effect is substrate specific, since no significant change was observed in halofantrine or vinblastine transport by this mutation, suggesting that it is important for substrates which bind to the TM11 binding pocket (described below), as demonstrated for QN transport [37].

### PfMDR1 drug-binding pocket

Transmembrane regions in ABC transporters are involved in ligand docking. Among the five naturally occurring functional PfMDR1 polymorphisms involved in antimalarial resistance, two are located in TM11, residues 1034 and 1042 (Figure 1D). Polymorphisms localised in this region of the human homologous transporter Pgp interfere with drug transport, suggesting the existence of a drug-binding pocket in this region [49].

In our model, the 1034/1042 and 86 positions co-localize in three dimension at a close region in the open state conformation of the protein (V shaped), whereas TM1 and TM11 come closer (Figure 1A and 1D). Spatial analysis shows that residue 86 in EL1 is in contact with the digestive vacuole (DV) lumen (Figure 1A–C), while the residues 1034 and 1042 alternate between facing the cytosol in the open state (V shaped, Figure 1A) and DV lumen in the closed state (Figure 1C).

**Figure 3:**
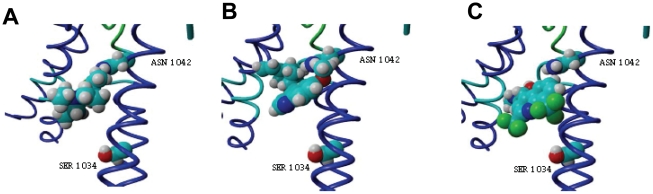
Docking of antimalarial in TM11. Docking of CQ (**A**), QN (**B**) and MQ (**C**) at residues 1042 and 1034 (serine and aspartic acid respectively) in TM11 is shown. The energies of docking for best pose were estimated to be −6.89 Kcal/mol for CQ, −7.86 Kcal/mol for QN and −5.69 Kcal/mol for MQ.

Several studies propose residues 1034 and 1042 as part of an antimalarial binding pocket [19], [39]. Using a refined model from the open state conformation we performed docking analysis in TM11 for several antimalarial drugs in order to characterize 1034 and 1042 residues as a drug binding site. Mefloquine (MQ), quinine (QN), and chloroquine (CQ) docked in the proposed binding site, preferentially interacting with residue 1042. The energies of docking for the best pose were estimated to be: −6.89 Kcal/mol for CQ (Figure 3A), −7.86 for QN (Figure 3B) and −5.69 for MQ (Figure 3C).

When residues 1042 and 1034 were mutated, the estimated docking capacity of binding was altered, strengthening the hypothesis that these residues are actively involved in the drug binding site. Introduction of a single mutation 1034C abolished the docking of MQ and QN but not CQ. CQ binds to the mutated TM11 (1034C) with an estimated energy of −7.55 Kcal/mol.

The single mutation of N1042D and the double mutation 1034C/1042D abolished the docking of all tested antimalarial.

### Cellular physiology of antimalarial transport by PfMDR1 and resistance

Together with PfMDR1, multidrug resistance in *P. falciparum* was shown to be highly associated with another gene, the chloroquine resistance transporter gene (pfcrt). Both transporters localize in the DV, with opposed directional flux characteristics. PfMDR1 is proposed to be a DV importer [50] whilst PfCRT was shown to be an antimalarial DV exporter [51]. To study the physiology of PfMDR1/PfCRT interference towards antimalarial resistance, we evaluate the contribution for antimalarial resistance of isogenic PfMDR1 mutant's clones with a CQ resistant (7G8) or sensitive (D10) genetic background (Table S1).

The target for antimalarial aminoquinoline is well known to be the parasite's DV. For this reason, chloroquine (CQ), amodiaquine (AQ) and desethylamodiaquine (DEAQ) were used as probe drugs. In order to study in exclusive the effect of PfMDR1 mutations, verapamil was used to block the PfCRT 76T variant.

To test the contribution of PfMDR1 for aminoquinoline we compared the index of resistance between parasites harbouring a wild type (SND) or resistant (CDY) haplotype at residues 1034, 1042 and 1246. These experiments were conducted in two different *pfcrt* genetic backgrounds carrying a 76T (7G8) or 76K allele (D10).

**Figure 4:**
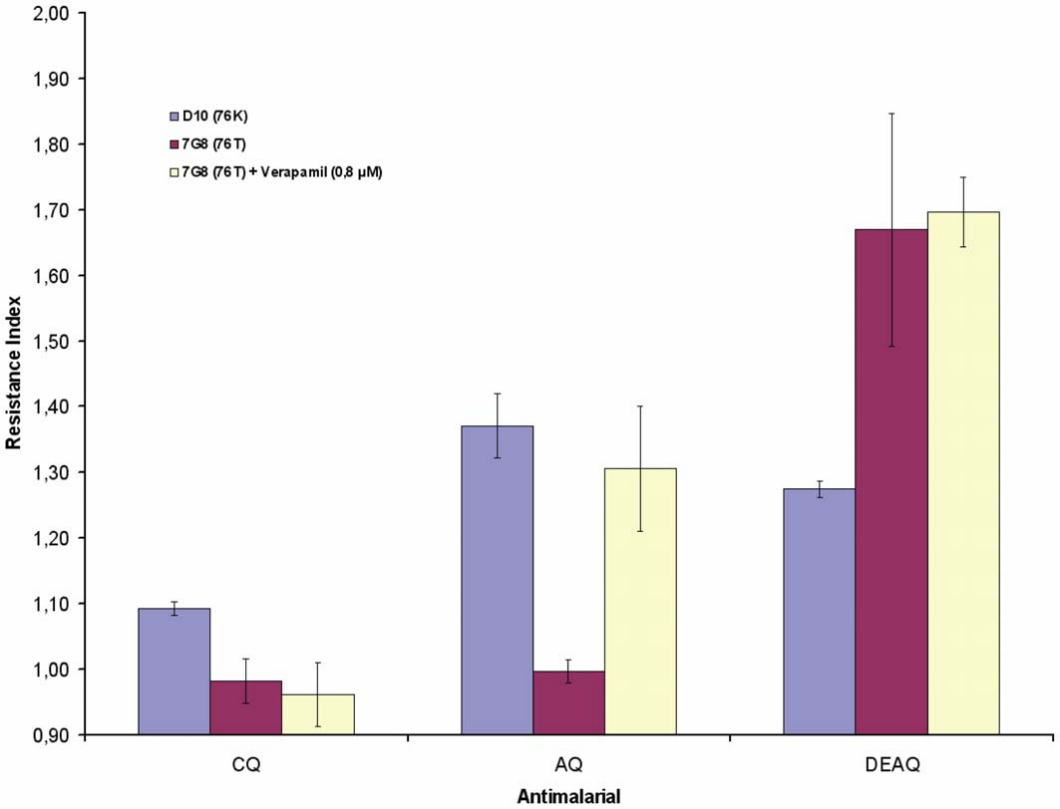
Contribution of PfMDR1 for antimalarial resistance. PfMDR1 Resistance Index (RI) was calculated, for each given antimalarial, as follow: for D10 - RI = D10^CDY^ EC_50_/D10^SND^ EC_50_ and equally for 7G8 - RI = 7G8^CDY^ EC_50_/7G8^SND^ EC_50_. Verapamil was used to block PfCRT.

Our results show that PfMDR1 contributed to resistance for all tested antimalarial. The differential effect of PfMDR1 for different aminoquinoline susceptibility was as follow: chloroquine<amodiaquine<desethylamodiaquine (Fig. 4). This association is related with the lipophobicity characteristics of the tested drugs and the PfCRT background. The corresponding Log *D* (pH 7.2) values for these drug are CQ:0.045<(DEAQ:1.183<AQ:2.60) (taken from [52]). In general, the involvement of PfMDR1 was stronger for AQ and DEAQ than the effect observed for CQ.

In the PfCRT 76K background (D10), the contribution of PfMDR1 was observed even for CQ while in 7G8 clone (76T) is only detected for DEAQ. Although, when PfCRT is blocked with verapamil in 7G8 clone, a significant increase is observed also for AQ to levels comparable with D10 index for AQ (Fig. 4).

These observations support the hypothesis that PfMDR1 is a vacuolar importer. In the presence of the mutant PfMDR1 (blocking antimalarial DV import), sensitivity to aminoquinoline is driven by its capacity to passively enter the DV membrane, demonstrated here through the correlation between the PfMDR1 resistance index and aminoquinoline lipophobicity characteristics.

Interestingly, taken together with the inverse association of aminoquinoline lipophobicity pattern for PfCRT-based resistance, an explanation may present itself for the *in vivo* co-selection of opposed functional haplotypes (highly effective PfCRT efflux and deficient PfMDR1 influx transporters) to reduce the dynamics of aminoquinoline accumulation in the DV, especially for hydrophobic compounds.

## Discussion

In the present work we propose a model for PfMDR1 structure and motion during transport. The existence of extensive literature describing the functional and molecular epidemiologic impact of polymorphisms in this transporter together with knowledge of the structure of ABC transporters, made it possible to predict the functional role of mutant residues in PfMDR1.

Our structural analyses, in the light of previous allelic exchange and transport kinetics studies [18], [19], [37], [39], demonstrate the existence of an internal allosteric modulation of protein transporting capacity by the three key polymorphisms herein studied. Accordingly, PfMDR1 variant 86Y/184Y/1246Y relates to a PfMDR1 low-performance quinoline antimalarial transporter. The inverse variant (86N/184F/1246D) relates to a higher-performance PfMDR1 transporter, being coherently associated with gene copy number amplification and its effects in terms of enhanced resistance to MQ and QN [7], [53].

The two most common PfMDR1 selected variants observed in African parasite populations harbour 86N/184F/1246D or 86Y/184Y/1246Y [8], [15], [20], [28]. Hence, the suggested altered allosteric control of transporter activity proposed by the model seems to be the main molecular phenotype associated with the influence of PfMDR1 in African parasites.

We describe that residues in TM11 (1034 and 1042) are related to phenotype modulation by altering a drug pocket in PfMDR1. The contact of residue 86 with the end of TM11 may explain an observed change in drug specificity through the alteration of PfMDR1 drug pocket conformation [37]. Polymorphisms in TM11 are geographically fixed in Asia and South America. This observation suggests that different genetic background/environment defines the fixation of a particular mechanism for PfMDR1 functional modulation.

From a mechanistic perspective, the haplotype encoding 86N, 1034S, 1042N and 1246D residues is related to a functional PfMDR1 importer into the DV [50]. Again, field observations show this haplotype to be related to mefloquine and lumefantrine resistance [7], [8], [20], [53]. This importer variant is oppositely related (promotes sensitivity) to CQ and amodiaquine (AQ) which acts within the DV [15], [28]–[31].

From a dynamic perspective, PfCRT antimalarial vacuolar efflux may affect the contribution of PfMDR1 to resistance. Supporting the hypothesis of a vacuolar antimalarial accumulation dynamic through PfCRT/PfMDR1 interaction, is the natural selection of functionally inversed polymorphisms of PfCRT and PfMDR1. PfCRT 76T promotes vacuolar efflux [54], [55] and is associated with PfMDR1 86Y (abrogates quinolines transport) [37]. Both forms are selectively associated with, as for example, CQ and AQ malaria chemotherapy [15], [28]–[31]. Oppositely, PfCRT 76K with PfMDR1 86N are associated with selection by aminoalcohol antimalarial drugs, such lumefantrine [8], [56].

Based on different genetic backgrounds, PfCRT/PfMDR1 dynamics can be either pro- or con- tolerance as has been previously reported to occur in a compensatory manner depending on the mechanism of action of the antimalarial [57].

We previously reported the importance of natural selection of different variants of PfMDR1 with different antimalarial chemotherapies [8], [15], [20], [28]. Now we describe how residues in PfMDR1 may be involved in drug resistance using a structural model.

In conclusion, we describe a model for structural changes associated with transport motion and we suggest the presence of a drug binding pocket in PfMDR1. Our results support recent findings suggesting this transporter as being a vacuolar antimalarial importer and propose a structural basis for the importance of residues 86, 184, 1034, 1042 and 1246 for drug specificity and how mutations at these residues may interfere with the composition of a drug pocket in TM11. The proposed models are expected to contribute to the prediction of the effects of other less studied PfMDR1 SNPs, while being a potential tool for the design of antimalarial drugs targeting this essential *P. falciparum* transporter.

## Materials and Methods

### Parasites and drug susceptibility assays

Four *P. falciparum* clones in which the *pfmdr1* 1034, 1042 and/or 1246 loci have been modified through allelic exchange [18] were selected for this study. They were obtained from the Malaria Research and Reference Reagent Resource Center (MR4©). Two clones were derived from the CQ sensitive D10 clone from Papua New Guinea i.e MRA-563: D10 *pfmdr1* SND (autologous transfectant) (D10^SND^) and MRA-565: D10 *pfmdr1* CDY (D10^CDY^). Two clones were derived from the CQ resistant clone 7G8 from Brazil i.e MRA-566: 7G8 *pfmdr1* SND (7G8^SND^) and MRA-567: 7G8 *pfmdr1* CDY (autologous transfectant). The clones were defrosted, adapted to continuous culture in supplemented RPMI-1640 and 5% haematocrite and then synchronized with sorbitol, according to established protocols.

The influence of the *pfmdr1* 1034C, 1042D and/or 1246Y SNPs on parasite response to CQ, AQ and DEAQ was determined with an HRP2-ELISA based assay *in vitro* as previously described [58]. *In vitro* cultured parasites were diluted to an initial parasitemia of 0.05% and aliquoted into microculture 96-well plates pre-dosed with ascending concentrations of 0–404 nM CQ, 0–67 nM AQ or 0–156 nM DEAQ. We also added VP 0.8 µM to a parallel setup.

After incubation at 37°C for 72 h, the samples were freeze-thawed, transferred and processed in pre-coated ELISA plates (Cellabs, Australia) for spectrophotometric analysis (Multiskan EX, Thermo Labsystems®, Helsingfors, Finland) of HRP2 produced during parasite growth. The IC50 and values were determined using HN-NonLin V1.05 Beta © H. Noedl 2001 (http://malaria.farch.net).

### Modelling

Protein sequences and structures from bacterial MsbA 3B5w (open-apo with inverted V shape), 3B5x (closed-apo) and 3B60 (open-apo with V shape) upon nucleotide-bind were downloaded from PDB database (www.pdb.org). PfMDR1 sequence is deposited in NCBI Protein database with accession number XP_001351787.

Sequences were aligned with blosum62 matrix in MultiAlin server [59] with manual refinement. Since MsbA is formed by a homodimer, PfMDR1 sequence was analyzed dividing sequence protein in the two symmetric halves and terminals trimmed to the homologous TMD and NBD. Residues 37–642, corresponding to TMD1 and NBD1 and residues 763–1400 corresponding to TMD2 and NBD2, were used in the alignment.

Alignment was then analyzed with Ali2D software (http://toolkit.tuebingen.mpg.de/ali2d), developed by the Department of Protein Evolution, Max-Planck-Institute for Developmental Biology (Germany) for sequence identity and secondary structure similarities. Secondary structure was determined with Psipred algorithm [60] and aminoacids group coloured with Mview [61].

Models were generated using Modeller software [62]. For structures modelling and analysis, divergent residues 479–486 and 496–531 in NBD1, IH4 987–998 and residues 1181–1227 were absent in the alignment. MaxSprout software at European Bioinformatics Institute server was used to generate protein backbone and side chain co-ordinates from the C-(alpha) trace [63]. The halves of the transporter were assembled by superimposition in the MsbA structures using the Mustang algorithm and adjusted manually [64]. Yasara [65] and WinCoot [66] software were used for visualization and refinement.

### N-Glycosylation sites prediction

Prediction of N-Glycosylation sites at the EL1 was performed using NetNGlyc 1.0 Server (http://www.cbs.dtu.dk/services/NetNGlyc/). The protein sequence including the predicted EL1 plus four boundary aminoacids comprising residues 79–91 was evaluated. A threshold of 0.5 was applied.

### Drug pocket modeling and docking

The model based in the 3B5w structure was further refined in WinCoot for docking studies. Docking was performed with Arguslab software [67]. Residues 1034 and 1042 in TM11 were defined as drug binding pockets and docking boxes defined as minimum volume for binding of 12.5×13×12.5 Å for CQ and QN and 13×13×13 Å for MQ were used. Best pose for the lowest energy of binding was considered. The CQ, QN and MQ structures were design and refined at PRODRG2 server [68].

## Supporting Information

Figure S1 **Protein sequences alignment of PfMDR1 and different MsbA structures.** The alignment describes the primary percentage of overall matching between PfMDR1 protein as well as secondary and tertiary structures features. Aminoacids letters are coloured by group identity default palette defined by Mview software. Background colour shows secondary structure, grey - coiled; brown- helix and yellow- beta-sheet. On top of the alignment is annotated the ABC conserved motifs as well as main structural characteristics: TM-transmembranes; EL- external loop; IH- internal helix. Stars identify functional residues in PfMDR1 transport: N86Y, Y184F, S1034C, N1042D and D1246Y.
(PDF)

Figure S2 **N-glycosylation sites prediction.** External loop 1 sequence (residues 79–91) was screened for putative N-glycosylation sites. Two asparagines localize in this loop at positions 84 and 86. Predictive potential sites obtained with NetNglyc 1.0 software.
(PDF)

Table S1 **PfCRT and PfMDR1 coding genotypes for clones D10^SND^, D10^CDY^, 7G8^SND^ and 7G8^CDY^.**
(PDF)

## References

[1] Wootton JC, Feng X, Ferdig MT, Cooper RA, Mu J (2002). Genetic diversity and chloroquine selective sweeps in Plasmodium falciparum.. Nature.

[2] Marsh K (1998). Malaria disaster in Africa.. Lancet.

[3] Trape JF, Pison G, Preziosi MP, Enel C, Desgrees du Lou A (1998). Impact of chloroquine resistance on malaria mortality.. C R Acad Sci III.

[4] White NJ (1999). Delaying antimalarial drug resistance with combination chemotherapy.. Parassitologia.

[5] WHO (2001). Antimalarial Drug Combination Therapy..

[6] Bhattarai A, Ali AS, Kachur SP, Martensson A, Abbas AK (2007). Impact of artemisinin-based combination therapy and insecticide-treated nets on malaria burden in Zanzibar.. PLoS Med.

[7] Price RN, Uhlemann AC, Brockman A, McGready R, Ashley E (2004). Mefloquine resistance in Plasmodium falciparum and increased pfmdr1 gene copy number.. Lancet.

[8] Sisowath C, Stromberg J, Martensson A, Msellem M, Obondo C (2005). *In vivo* selection of Plasmodium falciparum pfmdr1 86N coding alleles by artemether-lumefantrine (Coartem).. J Infect Dis.

[9] Noedl H, Se Y, Schaecher K, Smith BL, Socheat D (2008). Evidence of artemisinin-resistant malaria in western Cambodia.. N Engl J Med.

[10] Dondorp AM, Nosten F, Yi P, Das D, Phyo AP (2009). Artemisinin resistance in Plasmodium falciparum malaria.. N Engl J Med.

[11] Shen DW, Fojo A, Chin JE, Roninson IB, Richert N (1986). Human multidrug-resistant cell lines: increased mdr1 expression can precede gene amplification.. Science.

[12] Holland IB, Cole SPC, Kuchler K, Higgins CF (2003).

[13] Foote SJ, Thompson JK, Cowman AF, Kemp DJ (1989). Amplification of the multidrug resistance gene in some chloroquine-resistant isolates of *P. falciparum*.. Cell.

[14] Wilson CM, Serrano AE, Wasley A, Bogenschutz MP, Shankar AH (1989). Amplification of a gene related to mammalian mdr genes in drug-resistant Plasmodium falciparum.. Science.

[15] Holmgren G, Gil JP, Ferreira PM, Veiga MI, Obonyo CO (2006). Amodiaquine resistant Plasmodium falciparum malaria *in vivo* is associated with selection of pfcrt 76T and pfmdr1 86Y.. Infect Genet Evol.

[16] Sa JM, Twu O, Hayton K, Reyes S, Fay MP (2009). Geographic patterns of Plasmodium falciparum drug resistance distinguished by differential responses to amodiaquine and chloroquine.. Proc Natl Acad Sci U S A.

[17] Lopes D, Rungsihirunrat K, Nogueira F, Seugorn A, Gil JP (2002). Molecular characterisation of drug-resistant Plasmodium falciparum from Thailand.. Malar J.

[18] Reed MB, Saliba KJ, Caruana SR, Kirk K, Cowman AF (2000). Pgh1 modulates sensitivity and resistance to multiple antimalarials in Plasmodium falciparum.. Nature.

[19] Sidhu AB, Valderramos SG, Fidock DA (2005). pfmdr1 mutations contribute to quinine resistance and enhance mefloquine and artemisinin sensitivity in Plasmodium falciparum.. Mol Microbiol.

[20] Sisowath C, Ferreira PE, Bustamante LY, Dahlstrom S, Martensson A (2007). The role of pfmdr1 in Plasmodium falciparum tolerance to artemether-lumefantrine in Africa.. Trop Med Int Health.

[21] Chen N, Chavchich M, Peters JM, Kyle DE, Gatton ML (2010). De-amplification of pfmdr1-containing amplicon on chromosome 5 in Plasmodium falciparum is associated with reduced resistance to artelinic acid *in vitro*.. Antimicrob Agents Chemother.

[22] Chavchich M, Gerena L, Peters J, Chen N, Cheng Q (2010). Role of pfmdr1 amplification and expression in induction of resistance to artemisinin derivatives in Plasmodium falciparum.. Antimicrob Agents Chemother.

[23] Siarheyeva A, Sharom FJ (2009). The ABC transporter MsbA interacts with lipid A and amphipathic drugs at different sites.. Biochem J.

[24] Ward A, Reyes CL, Yu J, Roth CB, Chang G (2007). Flexibility in the ABC transporter MsbA: Alternating access with a twist.. Proc Natl Acad Sci U S A.

[25] Loo TW, Bartlett MC, Clarke DM (2008). Processing mutations disrupt interactions between the nucleotide binding and transmembrane domains of P-glycoprotein and the cystic fibrosis transmembrane conductance regulator (CFTR).. J Biol Chem.

[26] Oancea G, O'Mara ML, Bennett WF, Tieleman DP, Abele R (2009). Structural arrangement of the transmission interface in the antigen ABC transport complex TAP.. Proc Natl Acad Sci U S A.

[27] Dokomajilar C, Nsobya SL, Greenhouse B, Rosenthal PJ, Dorsey G (2006). Selection of Plasmodium falciparum pfmdr1 alleles following therapy with artemether-lumefantrine in an area of Uganda where malaria is highly endemic.. Antimicrob Agents Chemother.

[28] Holmgren G, Hamrin J, Svard J, Martensson A, Gil JP (2007). Selection of pfmdr1 mutations after amodiaquine monotherapy and amodiaquine plus artemisinin combination therapy in East Africa.. Infect Genet Evol.

[29] Kublin JG, Cortese JF, Njunju EM, Mukadam RA, Wirima JJ (2003). Reemergence of chloroquine-sensitive Plasmodium falciparum malaria after cessation of chloroquine use in Malawi.. J Infect Dis.

[30] Nsobya SL, Dokomajilar C, Joloba M, Dorsey G, Rosenthal PJ (2007). Resistance-mediating Plasmodium falciparum pfcrt and pfmdr1 alleles after treatment with artesunate-amodiaquine in Uganda.. Antimicrob Agents Chemother.

[31] Sutherland CJ, Alloueche A, Curtis J, Drakeley CJ, Ord R (2002). Gambian children successfully treated with chloroquine can harbor and transmit Plasmodium falciparum gametocytes carrying resistance genes.. Am J Trop Med Hyg.

[32] Schinkel AH, Kemp S, Dolle M, Rudenko G, Wagenaar E (1993). N-glycosylation and deletion mutants of the human MDR1 P-glycoprotein.. J Biol Chem.

[33] Gowda DC, Davidson EA (1999). Protein glycosylation in the malaria parasite.. Parasitol Today.

[34] Buchaklian AH, Funk AL, Klug CS (2004). Resting state conformation of the MsbA homodimer as studied by site-directed spin labeling.. Biochemistry.

[35] Loo TW, Bartlett MC, Clarke DM (2005). ATP hydrolysis promotes interactions between the extracellular ends of transmembrane segments 1 and 11 of human multidrug resistance P-glycoprotein.. Biochemistry.

[36] Taguchi Y, Kino K, Morishima M, Komano T, Kane SE (1997). Alteration of substrate specificity by mutations at the His61 position in predicted transmembrane domain 1 of human MDR1/P-glycoprotein.. Biochemistry.

[37] Sanchez CP, Rotmann A, Stein WD, Lanzer M (2008). Polymorphisms within PfMDR1 alter the substrate specificity for anti-malarial drugs in Plasmodium falciparum.. Mol Microbiol.

[38] Safa AR, Stern RK, Choi K, Agresti M, Tamai I (1990). Molecular basis of preferential resistance to colchicine in multidrug-resistant human cells conferred by Gly-185----Val-185 substitution in P-glycoprotein.. Proc Natl Acad Sci U S A.

[39] Lekostaj JK, Amoah LE, Roepe PD (2008). A single S1034C mutation confers altered drug sensitivity to PfMDR1 ATPase activity that is characteristic of the 7G8 isoform.. Mol Biochem Parasitol.

[40] Basco LK, Ringwald P (2002). Molecular epidemiology of malaria in Cameroon. X. Evaluation of PFMDR1 mutations as genetic markers for resistance to amino alcohols and artemisinin derivatives.. Am J Trop Med Hyg.

[41] Chaiyaroj SC, Buranakiti A, Angkasekwinai P, Looressuwan S, Cowman AF (1999). Analysis of mefloquine resistance and amplification of pfmdr1 in multidrug-resistant Plasmodium falciparum isolates from Thailand.. Am J Trop Med Hyg.

[42] Chen N, Russell B, Fowler E, Peters J, Cheng Q (2002). Levels of chloroquine resistance in Plasmodium falciparum are determined by loci other than pfcrt and pfmdr1.. J Infect Dis.

[43] Duraisingh MT, Jones P, Sambou I, von Seidlein L, Pinder M (2000). The tyrosine-86 allele of the pfmdr1 gene of Plasmodium falciparum is associated with increased sensitivity to the anti-malarials mefloquine and artemisinin.. Mol Biochem Parasitol.

[44] Price RN, Cassar C, Brockman A, Duraisingh M, van Vugt M (1999). The pfmdr1 gene is associated with a multidrug-resistant phenotype in Plasmodium falciparum from the western border of Thailand.. Antimicrob Agents Chemother.

[45] Zalis MG, Pang L, Silveira MS, Milhous WK, Wirth DF (1998). Characterization of Plasmodium falciparum isolated from the Amazon region of Brazil: evidence for quinine resistance.. Am J Trop Med Hyg.

[46] Carrier I, Gros P (2008). Investigating the role of the invariant carboxylate residues E552 and E1197 in the catalytic activity of Abcb1a (mouse Mdr3).. FEBS J.

[47] Loo TW, Bartlett MC, Clarke DM (2004). Processing mutations located throughout the human multidrug resistance P-glycoprotein disrupt interactions between the nucleotide binding domains.. J Biol Chem.

[48] Dalmas O, Orelle C, Foucher AE, Geourjon C, Crouzy S (2005). The Q-loop disengages from the first intracellular loop during the catalytic cycle of the multidrug ABC transporter BmrA.. J Biol Chem.

[49] Kajiji S, Talbot F, Grizzuti K, Van Dyke-Phillips V, Agresti M (1993). Functional analysis of P-glycoprotein mutants identifies predicted transmembrane domain 11 as a putative drug binding site.. Biochemistry.

[50] Rohrbach P, Sanchez CP, Hayton K, Friedrich O, Patel J (2006). Genetic linkage of pfmdr1 with food vacuolar solute import in Plasmodium falciparum.. EMBO J.

[51] Martin RE, Marchetti RV, Cowan AI, Howitt SM, Broer S (2009). Chloroquine transport via the malaria parasite's chloroquine resistance transporter.. Science.

[52] Bray PG, Hawley SR, Mungthin M, Ward SA (1996). Physicochemical properties correlated with drug resistance and the reversal of drug resistance in Plasmodium falciparum.. Mol Pharmacol.

[53] Price RN, Uhlemann AC, van Vugt M, Brockman A, Hutagalung R (2006). Molecular and pharmacological determinants of the therapeutic response to artemether-lumefantrine in multidrug-resistant Plasmodium falciparum malaria.. Clin Infect Dis.

[54] Sanchez CP, McLean JE, Rohrbach P, Fidock DA, Stein WD (2005). Evidence for a pfcrt-associated chloroquine efflux system in the human malarial parasite Plasmodium falciparum.. Biochemistry.

[55] Sanchez CP, McLean JE, Stein W, Lanzer M (2004). Evidence for a substrate specific and inhibitable drug efflux system in chloroquine resistant Plasmodium falciparum strains.. Biochemistry.

[56] Sisowath C, Petersen I, Veiga MI, Martensson A, Premji Z (2009). *In vivo* selection of Plasmodium falciparum parasites carrying the chloroquine-susceptible pfcrt K76 allele after treatment with artemether-lumefantrine in Africa.. J Infect Dis.

[57] Jiang H, Patel JJ, Yi M, Mu J, Ding J (2008). Genome-wide compensatory changes accompany drug- selected mutations in the Plasmodium falciparum crt gene.. PLoS One.

[58] Noedl H, Bronnert J, Yingyuen K, Attlmayr B, Kollaritsch H (2005). Simple histidine-rich protein 2 double-site sandwich enzyme-linked immunosorbent assay for use in malaria drug sensitivity testing.. Antimicrob Agents Chemother.

[59] Corpet F (1988). Multiple sequence alignment with hierarchical clustering.. Nucleic Acids Res.

[60] McGuffin LJ, Bryson K, Jones DT (2000). The PSIPRED protein structure prediction server.. Bioinformatics.

[61] Brown NP, Leroy C, Sander C (1998). MView: a web-compatible database search or multiple alignment viewer.. Bioinformatics.

[62] Sali A (1995). Modeling mutations and homologous proteins.. Curr Opin Biotechnol.

[63] Holm L, Sander C (1991). Database algorithm for generating protein backbone and side-chain co-ordinates from a C alpha trace application to model building and detection of co-ordinate errors.. J Mol Biol.

[64] Konagurthu AS, Whisstock JC, Stuckey PJ, Lesk AM (2006). MUSTANG: a multiple structural alignment algorithm.. Proteins.

[65] Krieger E, Koraimann G, Vriend G (2002). Increasing the precision of comparative models with YASARA NOVA–a self-parameterizing force field.. Proteins.

[66] Emsley P, Cowtan K (2004). Coot: model-building tools for molecular graphics.. Acta Crystallogr D Biol Crystallogr.

[67] Thompson MA http://www.arguslab.com.

[68] Schuttelkopf AW, van Aalten DM (2004). PRODRG: a tool for high-throughput crystallography of protein-ligand complexes.. Acta Crystallogr D Biol Crystallogr.

