# Supplementary material for: PfMDR1: Mechanisms of Transport Modulation by Functional Polymorphisms
Source: PLoS One. 2011 Sep 1;6(9):e23875. doi: 10.1371/journal.pone.0023875 (PMC3164660; doi:10.1371/journal.pone.0023875)
Supplement: Figure S1 — Protein sequences alignment of PfMDR1 and different MsbA structures. The alignment describes the primary percentage of overall matching between PfMDR1 protein as well as secondary and tertiary structures features. Aminoacids letters are coloured by group identity default palette defined by Mview software. Background colour shows secondary structure, grey - coiled; brown- helix and yellow- beta-sheet. On top of the alignment is annotated the ABC conserved motifs as well as main structural characteristics: TM-transmembranes; EL- external loop; IH- internal helix. Stars identify functional residues in PfMDR1 transport: N86Y, Y184F, S1034C, N1042D and D1246Y. (PDF) [file pone.0023875.s001.pdf]

Supplementary figure 1

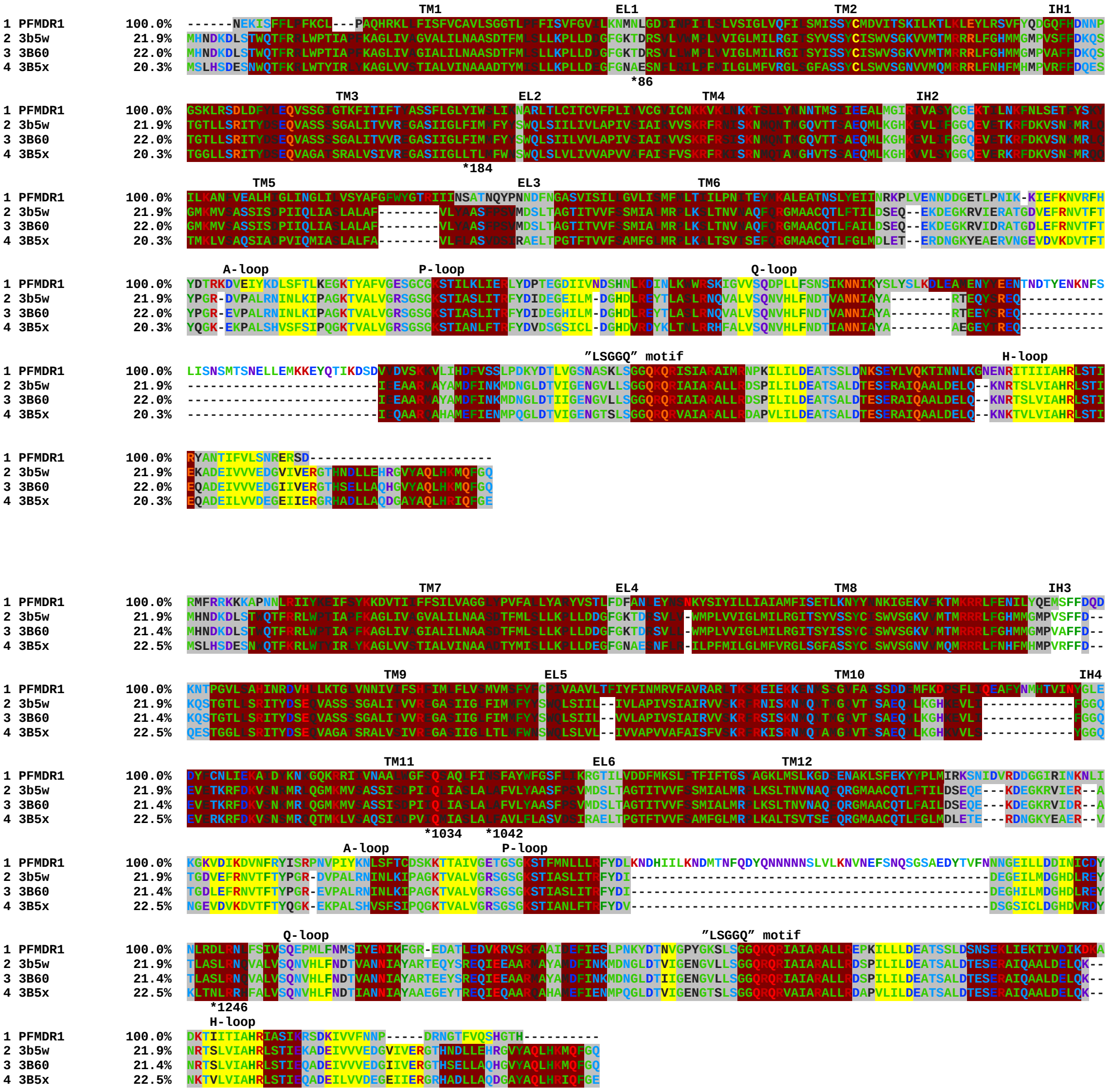

Protein sequences alignment of PfMDR1 and different MsbA structures.

The alignment describes the primary percentage of overall matching between PfMDR1 protein as well as secondary and tertiary structures features. Aminoacids letters are coloured by group identity default palette defined by Mview software. Background colour shows secondary structure, grey - coiled; brown- helix and yellow- beta-sheet.

On top of the alignment is annotated the ABC conserved motifs as well as main structural characteristics: TM-transmembranes; EL- external loop; IH- internal helix. Stars identify functional residues in PfMDR1 transport: N86Y, Y184F, S1034C, N1042D and D1246Y.
