# Supplementary material for: PfMDR1: Mechanisms of Transport Modulation by Functional Polymorphisms
Source: PLoS One. 2011 Sep 1;6(9):e23875. doi: 10.1371/journal.pone.0023875 (PMC3164660; doi:10.1371/journal.pone.0023875)
Supplement: Figure S2 — N-glycosylation sites prediction. External loop 1 sequence (residues 79–91) was screened for putative N-glycosylation sites. Two asparagines localize in this loop at positions 84 and 86. Predictive potential sites obtained with NetNglyc 1.0 software. (PDF) [file pone.0023875.s002.pdf]

Supplementary figure 2

```
Name:  Sequence       Length:  13
GVILKNMNLGDDI
.....n.n.....
79

(Threshold=0.5)
----------------------------------------------------------------------
SeqName      Position  Potential   Jury    N-Glyc
                                agreement  result
----------------------------------------------------------------------
Sequence        6 NMNL   0.5643     (8/9)    +
Sequence        8 NLGD   0.7344     (9/9)    ++
----------------------------------------------------------------------
```

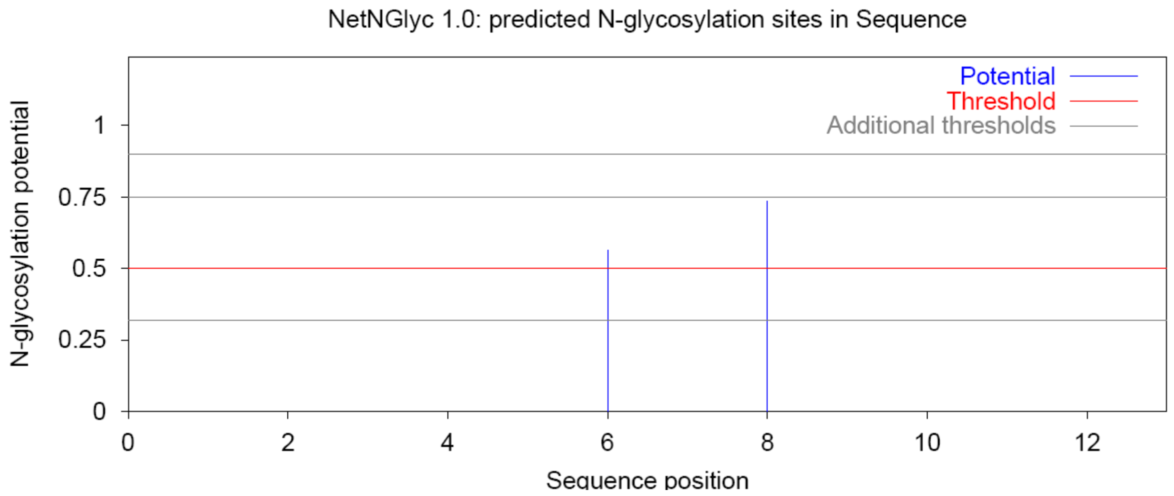

N-glycosylation sites prediction. External loop 1 sequence (residues 79-91) was screened for putative N-glycosylation sites. Two asparagines localize in this loop at positions 84 and 86. Predictive potential sites results from NetNglyc 1.0 software is shown.
