# Supplementary material for: PfMDR1: Mechanisms of Transport Modulation by Functional Polymorphisms
Source: PLoS One. 2011 Sep 1;6(9):e23875. doi: 10.1371/journal.pone.0023875 (PMC3164660; doi:10.1371/journal.pone.0023875)
Supplement: Table S1 — PfCRT and PfMDR1 coding genotypes for clones D10SND, D10CDY, 7G8SND and 7G8CDY. (PDF) [file pone.0023875.s003.pdf]

Table S1 - PfCRT and PfMDR1 coding genotypes for clones D10^{SND} , D10^{CDY}, 7G8^{SND} and 7G8^{CDY}.

**Clone**	**PfCRT**	**PfMDR1**				
	**aa**	**aa**				
	**76**	**86**	**184**	**1034**	**1042**	**1246**
D10^{SND}	K	N	Y	S	N	D
D10^{CDY}	K	N	Y	C	D	Y
7G8^{SND}	T	N	F	S	N	D
7G8^{CDY}	T	N	F	C	D	Y
